# Supplementary material for: High-throughput quantitation of SARS-CoV-2 antibodies in a single-dilution homogeneous assay
Source: Sci Rep. 2021 Jun 10;11:12330. doi: 10.1038/s41598-021-91300-5 (PMC8192771; doi:10.1038/s41598-021-91300-5)
Supplement: Supplementary file 1 — Supplementary Information. [file 41598_2021_91300_MOESM1_ESM.pdf]

Supplementary materials for

High-throughput quantitation of SARS-CoV-2 antibodies in a single-dilution homogeneous assay

Authors

Markus H. Kainulainen[1], Eric Bergeron[1], Payel Chatterjee[1], Asheley P. Chapman[2], Joo Lee[3], Asiya Chida[3], Xiaoling Tang[3], Rebekah E. Wharton[4], Kristina B. Mercer[5], Marla Petway[3], Harley M. Jenks[1], Timothy D. Flietstra[1], Amy J. Schuh[1], Panayampalli S. Satheshkumar[6], Jasmine M. Chaitram[7], S. Michele Owen[8], Laura K. McMullan[1], Mike Flint[1], M. G. Finn[2], Jason M. Goldstein[3], Joel M. Montgomery[1], Christina F. Spiropoulou[1]*

Affiliations:

[1] Viral Special Pathogens Branch, Division of High-Consequence Pathogens and Pathology, Centers for Disease Control and Prevention, 1600 Clifton Rd, Atlanta, GA 30329, USA

[2] School of Chemistry and Biochemistry, School of Biological Sciences, Georgia Institute of Technology, 901 Atlantic Dr., Atlanta, GA 30332, USA

[3] Reagent and Diagnostic Services Branch, Division of Scientific Resources, Centers for Disease Control and Prevention, 1600 Clifton Rd, Atlanta, GA 30329, USA

[4] Emergency Response Branch, Division of Laboratory Sciences, Centers for Disease Control and Prevention, 4770 Buford Hwy., Atlanta, GA 30341, USA

[5] Newborn Screening & Molecular Biology Branch, Division of Laboratory Sciences, Centers for Disease Control and Prevention, 4770 Buford Hwy., Atlanta, GA 30341, USA

[6] Poxvirus and Rabies Branch, Division of High-Consequence Pathogens and Pathology, Centers for Disease Control and Prevention, 1600 Clifton Rd, Atlanta, GA 30329, USA

[7] Division of Laboratory Systems, Centers for Disease Control and Prevention, 1600 Clifton Rd, Atlanta, GA 30329, USA

[8] National Center for HIV/AIDS, Viral Hepatitis, STD, and TB Prevention, Centers for Disease Control and Prevention, 1600 Clifton Rd, Atlanta, GA 30329, USA

* Corresponding author

CDC/H18-SB/SB153

1600 Clifton Rd

Atlanta, GA 30329, USA

ccs8@cdc.gov

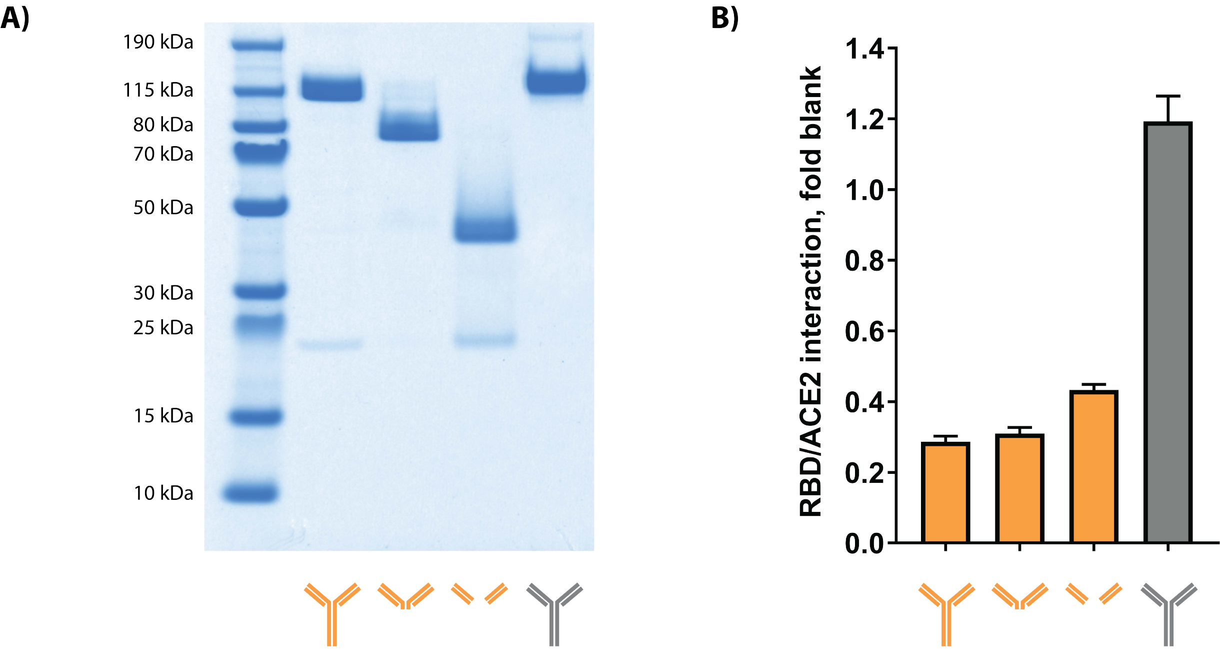

Supplementary Fig. 1: mAbs, F(ab')2 and Fab fragments analyzed by SDS-PAGE; RBD/ACE2 inhibition assay.

A) SARS-CoV-2 RBD-specific mAb 3A2 (orange) was analyzed with non-reducing, non-heated SDS-PAGE, both intact and after cleavege to F(ab')2 and Fab fragments as described in Materials and Methods. A non-targeting isotype control mAb (gray) was analyzed in parallel.

B) The ability of 3A2 mAb, 3A2 F(ab')2, 3A2 Fab, and isotype control mAb to inhibit the interaction between SARS-CoV-2 RBD and its receptor ACE2 was quantified using the assay described in Materials and Methods.

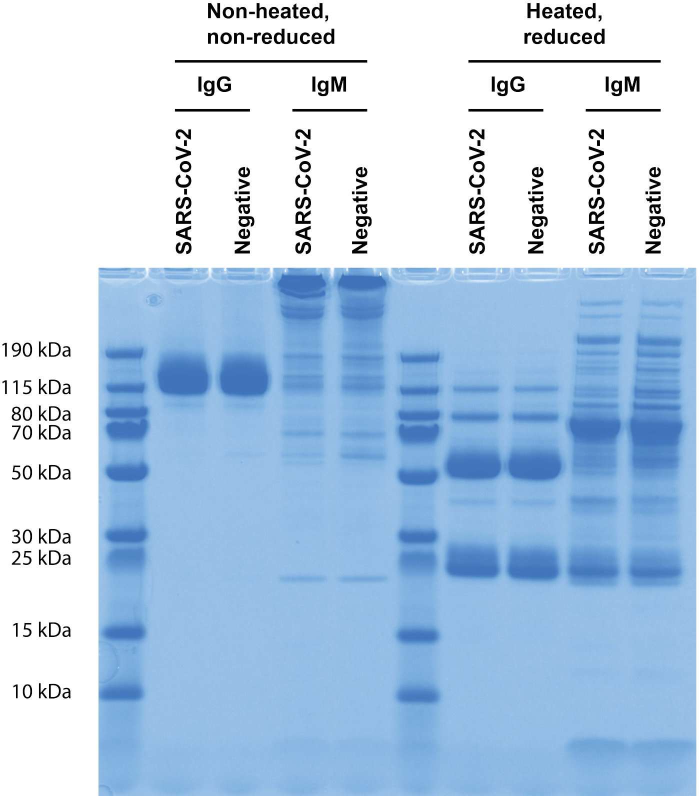

Supplementary Fig. 2: IgG and IgM antibodies purified from pooled sera of convalescent Covid-19 patients or from a control serum pool. The purification products (10 µg/lane) were separated by SDS-PAGE on a 4–12% Bis-Tris gel with MES running buffer and stained with InstantBlue Coomassie stain.

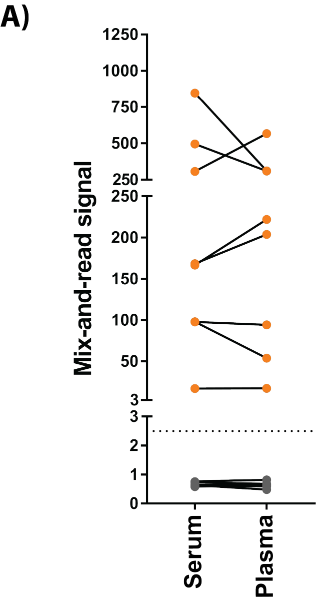

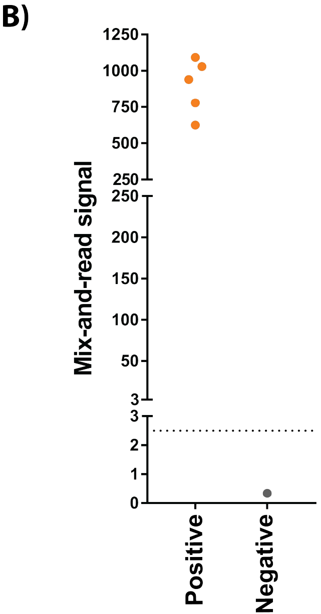

Supplementary Fig. 3: Comparing serum and plasma as sample matrices; analyzing mouse serum.
A) Paired human serum and EDTA plasma samples from 8 SARS-CoV-2-positive and 8 negative individuals were obtained from commercial sources and tested in parallel by the mix-and-read assay. Orange: samples from individuals with history of PCR-confirmed SARS-CoV-2 infection. Gray: negative control samples.
B) Analyzing mouse serum. Five serum samples from mice that were repeatedly immunized in order to generate antibodies against SARS-CoV-2 spike domains were analyzed as positive controls, and negative control serum was from an untreated mouse.

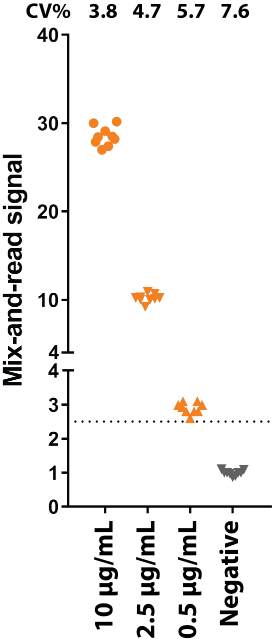

Supplementary Fig. 4: Run-to-run reproducibility of quantification controls. 3A2 mAb was spiked into normal human serum in 3 different concentrations, or serum was left without mAb for a negative control. Aliquots were stored frozen and run together with samples of interest to monitor day-to-day and run-to-run performance of the assay. Data from 9 independent runs presented. CV; coefficient of variation.

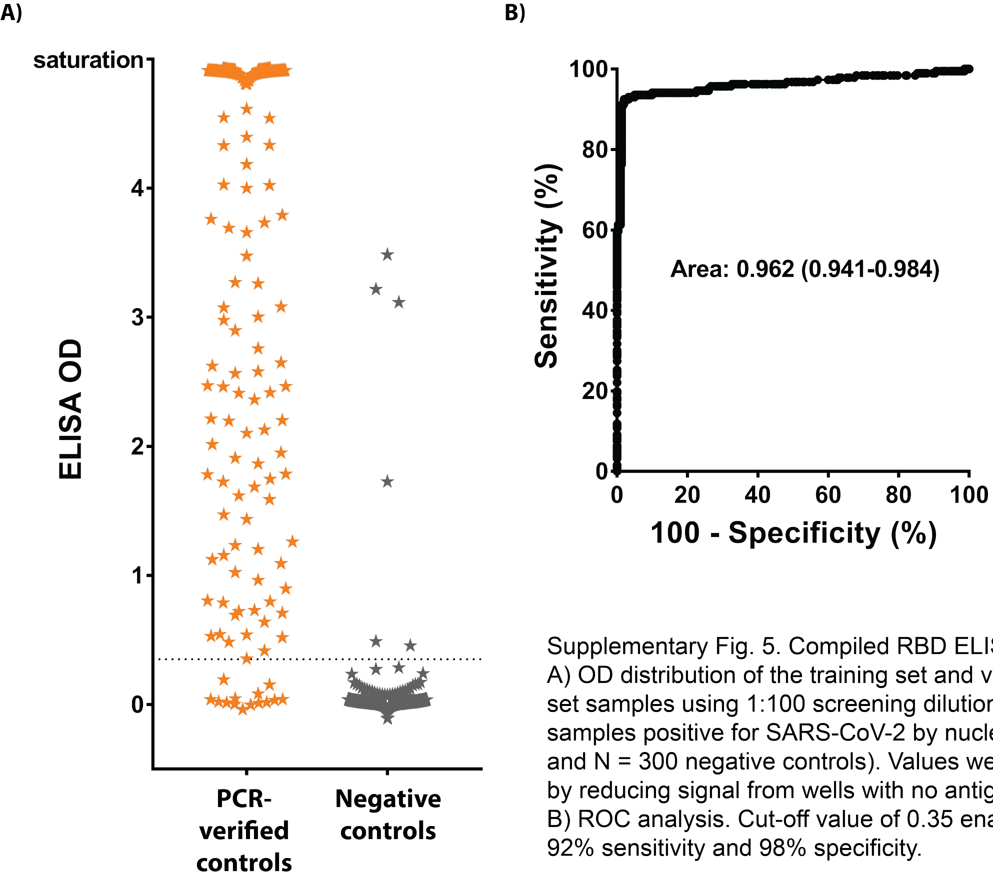

Supplementary Fig. 5. Compiled RBD ELISA results.
A) OD distribution of the training set and validation set samples using 1:100 screening dilution (N = 186 samples positive for SARS-CoV-2 by nucleic acid testing and N = 300 negative controls). Values were corrected by reducing signal from wells with no antigen.
B) ROC analysis. Cut-off value of 0.35 enables 92% sensitivity and 98% specificity.
